# Early modifications of the gut microbiome in children with hepatic sinusoidal obstruction syndrome after hematopoietic stem cell transplantation

**DOI:** 10.1038/s41598-021-93571-4

**Published:** 2021-07-12

**Authors:** Riccardo Masetti, Elena Biagi, Daniele Zama, Edoardo Muratore, Federica D’Amico, Davide Leardini, Silvia Turroni, Arcangelo Prete, Patrizia Brigidi, Andrea Pession

**Affiliations:** 1 Pediatric Oncology and Hematology “Lalla Seràgnoli”, Pediatric Unit, IRCCS Azienda Ospedaliero-Universitaria di Bologna, 40138 Bologna, Italy; 2 Unit of Microbiome Science and Biotechnology, Department of Pharmacy and Biotechnology, University of Bologna, 40126 Bologna, Italy; 3 Department of Medical and Surgical Sciences (DIMEC), University of Bologna, 40138 Bologna, Italy

**Keywords:** Haematopoietic stem cells, Paediatric cancer, Cancer therapy

## Abstract

Hepatic sinusoidal obstruction syndrome (SOS/VOD) represents a dramatic complication of hematopoietic stem cell transplantation (HSCT), particularly in children. Recent evidence has suggested a role for the gut microbiome (GM) in the context of HSCT and its related complications, but no data are available on the relationship between GM and SOS/VOD. Here, we conducted a retrospective case–control study in allo-HSCT pediatric patients developing or not SOS/VOD and profiled their GM over time, from before the transplant up to 72 days after. A rich and diverse GM before HSCT was found to be associated with a reduced likelihood of developing SOS/VOD. Furthermore, prior to transplant, patients not developing SOS/VOD showed an enrichment in some typically health-associated commensals, such as *Bacteroides*, *Ruminococcaceae* and *Lachnospiraceae*. Their levels remained overall higher until post-transplant. This high-diversity configuration resembles that described in other studies for other HSCT-related complications, including graft-versus-host disease, potentially representing a common protective GM feature against HSCT complications.

## Introduction

Hepatic sinusoidal obstruction syndrome (SOS), also known as veno-occlusive disease (VOD) is a potentially life-threatening complication of hematopoietic stem cell transplantation (HSCT). It is caused by the damage generated during the conditioning regimen to sinusoidal endothelial cells and hepatocytes in zone 3 of the hepatic acinus^1^. Pediatric patients are at higher risk of developing SOS/VOD rather than adults, showing an incidence of approximately 20–30%, compared to 9–14% in adults^2,3^. In a large pediatric cohort, the survival probability at 1 year for patients with or without severe and very severe SOS/VOD was 61% and 77%, respectively^3^.

Mounting evidence has suggested that the gut microbiome (GM) is associated with HSCT outcomes^4^and may play a pivotal role in the pathophysiology of the major HSCT complications in children, namely bloodstream infection (BSI) and graft-vs-host disease (GvHD)^5,6^. In particular, it is well known that the GM can act as a reservoir for BSI pathogens^5^ and pre-transplant GM signatures predicting outcomes such as subsequent GvHD occurrence have been consistently identified^6,7^, These signatures include lower diversity, increased proportions of pathogens or pathobionts, and reduced amounts of beneficial microbes, generally producers of short-chain fatty acids (SCFAs), which are key regulators of metabolic and immunological homeostasis^8^. To date, no information is available on the relationship between GM and SOS/VOD. However, preclinical studies suggest that microbial products translocated across impaired mucosal barriers may participate in the pathogenesis of endothelial damage, interfering with procoagulant and fibrinolytic endothelial responses^9,10^. This applies to lipopolysaccharide (LPS), a bacterial endotoxin that activates various signaling mechanisms in endothelial cells, ultimately leading to cellular dysfunction and injury^11^, as well as to other molecules directly produced or contributed by GM, such as tryptophan metabolites, trimethylamine-N-oxide and SCFAs. While the first two are recognized to have prothrombotic activity^12,13^, SCFAs are well-known anti-inflammatory metabolites that have also been shown to have hypotensive effects through binding to Gpr41 receptor, thus possibly improving circulation^14^.

In an attempt to shed some light on the possible relationship between GM and SOS/VOD, here we conducted a retrospective case–control study in allo-HSCT pediatric patients who developed SOS/VOD compared to control patients who did not develop this complication.

## Methods

### Patients enrollment

We performed a retrospective single-institution case–control study at the pediatric HSCT Unit of the University of Bologna, from 01/01/2015 to 31/12/2019. This study was approved by the Ethics Committee (ref. number 19/2013/U/Tess) and written informed consent was obtained from each enrolled patient or parent/legal guardian. Study inclusion criteria were the availability of a pre-HSCT fecal sample and of at least two samples collected after HSCT. Allo-HSCT pediatric recipients were selected among previously enrolled patients^6,15^. Patients in the control group were matched 1:1 by carefully matching for several microbiome-associated and SOS/VOD-related confounding factors^16^, such as age, sex, type of disease, conditioning regimen, antibiotic prophylaxis and type of nutrition, as well as source of stem cells. SOS/VOD grading was calculated according to the new EBMT (European Society for Blood and Marrow Transplantation) criteria for the diagnosis and grading of pediatric SOS/VOD^17^. All the patients developing SOS/VOD received defibrotide from the date of diagnosis for at least 21 days until resolution of signs and symptoms of SOS/VOD. When febrile neutropenia occurred, ceftazidime was used as first-line antibiotic therapy. Fecal samples were collected before and after transplant, up to 72 days post-HSCT, and stored at − 80 °C until analysis.

### Microbial DNA extraction and sequencing

Microbial DNA was extracted from about 250 mg of stool sample using the repeated bead-beating plus column method, as previously described^6,18^. The V3–V4 hypervariable region of the 16S rRNA gene was amplified using primers 341F and 785R with Illumina overhang adapter sequences. In addition to being considered among the best options for reliable taxonomic inference^19^, the V3–V4 region was chosen for comparative purposes, as some sequences had already been generated using primers targeting this region. PCR products were purified using a magnetic bead-based system (Agencourt AMPure XP; Beckman Coulter, Brea, CA) and indexed by limited-cycle PCR using Nextera technology. Indexed libraries were further cleaned up as above and pooled at equimolar concentration. The final library was denatured and diluted to 5 pmol/l with a 20% PhiX control. Sequencing was performed on an Illumina MiSeq platform using the 2 × 250 bp paired-end protocol, per manufacturer's instructions (Illumina, San Diego, CA). Raw sequence reads were deposited in MG-RAST (https://www.mg-rast.org/linkin.cgi?project=mgp97083).

### Bioinformatics and statistics

The bioinformatic processing of the sequencing reads was performed using a pipeline combining PANDAseq^20^ and QIIME 2^21^ as recently described^22^. Briefly, length and quality-filtered reads were clustered into Amplicon Sequence Variants (ASVs) with DADA2^23^. Singleton ASVs and chimeras were discarded during analysis. The taxonomy was assigned using the vsearch classifier^24^ against the Greengenes database as a reference (release May 2013). Alpha diversity was calculated using several metrics, such as the number of observed ASVs, the Shannon index, the Chao1 index, the inverse Simpson index and the phylogenetic metrics, Faith’s PD index, phylogenetic entropy (PE) and the abundance weighted evolutionary distinctiveness (AEDt) index. Beta diversity was estimated based on weighted and unweighted UniFrac distances and visualized on a Principal Coordinates Analysis (PCoA) plot.

All statistical analysis was performed in R using the packages vegan^25^, MADE4^26^, adiv^27^ and mallorn^28^. The significance of the separation between study groups in the PCoA space was assessed by a permutation test with pseudo-F ratio using the function adonis in vegan. The bacterial genera most contributing to the ordination space were identified using the function envfit of vegan. Differences between groups for both taxonomic profile and alpha diversity were evaluated by Wilcoxon test. P values were corrected for multiple comparisons using the Benjamini–Hochberg method when appropriate. A P value ≤ 0.05 was considered statistically significant, while a P value ≤ 0.1 a tendency. Samples were grouped according to the sampling date relative to HSCT as previously reported^6,18^, i.e., “PRE” (samples taken before HSCT within a maximum of 30 days before HSCT), “HSCT” (samples taken up to 30 days after HSCT), and “POST” (samples taken more than 30 days after HSCT).

### Quantitative PCR analysis

Quantitative PCR (qPCR) was used to confirm trends in taxa of interest, as emerged from the UniFrac-based PCoA analysis and the reconstruction of the longitudinal GM profiles. Specifically, we used a primer set targeting the 16S rDNA of *Bacteroides*^29^, and two primer sets for the 16S rDNA of *Lachnospiraceae* and *Ruminococcaceae* genera^30,31^. For the qPCR assays, genomic DNA was diluted with PCR-grade water (Hoffmann-La Roche, Basel, Switzerland) to a final concentration of 5 ng/µl. Reaction mixtures were prepared in a total volume of 20 μl using SYBR Select PCR master mix (Thermo Fisher Scientific, Waltham, MA), 0.2 μmol/l of each primer and 2 µl of diluted DNA. Amplification was performed on a StepOne Real-Time PCR System instrument (Thermo Fisher Scientific) with an initial denaturation step at 95 °C for 10 min, followed by 40 cycles of denaturation at 95 °C for 15 s, annealing at 55–61 °C for 30–60 s and elongation at 72 °C for 45 s, with a final elongation step at 72 °C for 5 min. For the annealing temperature and time for each primer set, see the original papers^30,31^. Melting curves for PCR product identification were obtained immediately after amplification. Standard curves were obtained by amplification of DNA from *Bacteroides eggerthii* DSM 20697 and *Coprococcus comes* ATCC 27758. Results were expressed as log10 16S rRNA gene copies/ng of DNA. Differences between and within groups were evaluated by Wilcoxon test.

### Ethics declarations

This study was approved by the IRCCS Azienda Ospedaliero-Universitaria di Bologna ethics committee (ref. number 19/2013/U/Tess). Written informed consent was obtained from each enrolled patient or parent/legal guardian. The study was conducted in accordance with the Declaration of Helsinki and Good Clinical Practice guidelines.

## Results

### Patients characteristics

Eighteen allo-HSCT pediatric recipients were selected among previously enrolled patients (Table 1). Nine patients developed severe or very severe SOS/VOD and nine patients were matched as controls (see Supplemental Tables S1–S4 for the distribution of microbiome-associated and SOS/VOD-related confounding factors, see Table 1 footnotes for exact matching). The cumulative incidence of SOS/VOD in our center was 15.38%. Three patients received haplo-HSCT with post-transplant cyclophosphamide. None of the patients received either Gemtuzumab or Inotuzumab prior to HSCT, or total body irradiation-based therapy in the conditioning regimen. A detailed description of antibiotic exposure is given in Table 2.

**Table 1:** Clinical features of enrolled patients.

ID	Age at HSCT, years	Time from diagnosis to HSCT, years	Time from last hospitalization, days	Gender	Diagnosis	Complete remission at transplant	Conditioning regimen (ATG)	Source of HSC	Type of Transplant	VOD	Day of VOD diagnosis	VOD grade	BSI (day)	GvHD grade (day)	EN
#1	16.6	0.3	108	M	MDS	No	BUS + EDX + L-PAM (ATG)	PBSC	MUD	Yes	10	3	No	I (+ 20)	No
#2	8.5	0.8	51	F	ALL	1st CR	BUS + THIO + EDX (ATG)	BM	MUD	Yes	19	3	No		Yes
#3	14.3	2.3	n.e.	F	ALL	2nd CR	BUS + THIO + EDX (ATG)	BM	MUD	Yes	18	4	No		Yes
#4	9.3	10.0	11	F	TM	No	BUS + THIO + FLUDA (ATG)	BM	MUD	Yes	17	4	*E. coli* (+ 8)		No
#5	5.5	2.5	109	M	ALL	2nd CR	THIO + TREO + FLUDA	BM	Haploidentical	Yes	26	4	No	III–IV (+ 22)	No
#6	18.2	0.5	19	M	AML	1st CR	BUS + EDX + L-PAM (ATG)	PBSC	MUD	Yes	21	3	No		Yes
#7	3.0	0.8	69	F	MDS	No	BUS + EDX + L-PAM (ATG)	BM	MUD	Yes	15	4	No		Yes
#8	1.0	0.4	5	F	ALL	1st CR	BUS + THIO + EDX	BM	MFD	Yes	15	4	No	II (+ 35)	No
#9	1.3	0.4	13	M	AML	1st CR	BUS + EDX + L-PAM	BM	MFD	Yes	25	3	No		No
#10	4.6	0.8	32	F	ALL	2nd CR	BUS + THIO + EDX (ATG)	BM	MUD	No			No		Yes
#11	20.6	0.8	93	M	ALL	1st CR	BUS + THIO + EDX (ATG)	BM	MUD	No			No	II (+ 15)	No
#12	8.1	5.2	15	M	ALL	3rd CR	BUS + THIO + FLUDA	BM	Haploidentical	No			No		Yes
#13	10.5	3.8	133	F	AML	2nd CR	THIO + TREO + FLUDA	BM	MFD	No			*S. epidermidis*, *E. faecalis *(+ 6)		No
#14	15.9	1.2	18	F	AML	2nd CR	BUS + THIO + FLUDA	BM	Haploidentical	No			No	II (+ 36)	Yes
#15	0.9	0.6	69	F	ALL	1st CR	BUS + THIO + EDX (ATG)	BM	MUD	No			*E. coli* (+ 2)		No
#16	6.0	4.4	12	M	TM	No	BUS + THIO + FLUDA (ATG)	BM	MUD	No			No	II (+ 26)	No
#17	14.4	0.3	48	F	AML	2nd CR	BUS + EDX + L-PAM (ATG)	BM	MUD	No			*E. coli*, *E. faecium*, *S. warneri *(+ 5)	I (+ 23)	Yes
#18	2.2	0.4	150	F	JMML	No	BUS + EDX + L-PAM (ATG)	BM	MUD	No			*S. epidermidis*, *S. vestibularis *(+ 36)	I (+ 35)	Yes

*ALL *acute lymphoblastic leukemia, *AML *acute myeloid leukemia, *BM *bone marrow, *BSI *blood stream infections, *BUS *busulfan, *CR *complete remission, *EDX *cyclophosphamide, *EN *enteral nutrition, *FLUDA *fludarabine, *GvHD *graft versus host disease, *HSC *hematopoietic stem cell, *HSCT *hematopoietic stem cell transplantation, *JMML *juvenile myelomonocytic leukemia, *L-PAM *melphalan, *MDS *myelodysplastic syndrome, *MFD *matched familiar donor, *MUD *matched unrelated donor, *PBSC *peripheral blood stem cells, *THIO *thiotepa, *TM *thalassemia major, *VOD *veno-occlusive disease. Patient’s matching was performed as following: #1–#17; #2–#14; #3–#12; #4–#16; #5–#13; #6–#11; #7–#10; #8–#15; #9–18.

**Table 2:** List of antibiotics used for each patient during hospitalization. SOS/VOD group.

ID	Levofloxacin prophylaxis	Antibiotic used during transplant	Total days of antibiotic
#1	No	Ceftazidime from + 4 to + 15. Piperacillin–Tazobactam from + 16 to + 23	20
#2	No	Ceftazidime from + 6 to + 18. Ceftazidime from + 36 to + 46	24
#3	No	Ceftazidime from + 5 to + 9. Piperacillin–Tazobactam from + 9 to + 17. Teicoplanin from + 15 to + 19. Meropenem from + 17 to + 26. Ceftazidime from + 26 to + 31. Piperacillin–Tazobactam from + 34 to + 48	38
#4	Yes	Levofloxacin from − 9 to − 6. Ceftazidime from + 5 to + 17. Teicoplanin from + 6 to + 19. Piperacillin–Tazobactam from + 20 to + 28	28
#5	Yes	Levofloxacin from − 9 to − 6. Ceftazidime from − 5 to + 2. Ceftazidime from + 6 to + 8. Piperacillin–Tazobactam from + 8 to + 21. Ceftazidime from + 21 to + 34. Ceftazidime from + 146 to + 153	49
#6	No	Ceftazidime from + 3 to + 5, Piperacillin–Tazobactam from + 5 to + 21. Meropenem from + 21 to + 45. Piperacillin–Tazobactam from + 45 to + 60. Linezolid from + 19 to + 36	58
#7	No	Piperacillin–Tazobactam from + 4 to + 10. Teicoplanin from + 8 to + 10. Daptomycin from + 10 to + 16. Meropenem from + 10 to + 21	18
#8	Yes	Levofloxacin from − 9 to + 14. Ceftazidime from + 1 to + 17	27
#9	Yes	Levofloxacin from − 9 to + 14. Ceftazidime from + 6 to + 14. Ceftazidime from + 25 to + 36. Teicoplanin from + 26 to + 36	36
#10	No	Piperacillin–Tazobactam from + 5 to + 9. Vancomycin from + 8 to + 11. Meropenem from + 9 to + 19. Linezolid from + 11 to + 19. Ceftazidime from + 22 to + 26. Clindamycin from + 26 to + 32. Piperacillin–Tazobactam from + 26 to + 32	26
#11	No	Meropenem from + 7 to + 18. Vancomycin from + 7 to + 18	12
#12	No	Ceftazidime from + 5 to + 20. Ceftazidime from + 32 to + .47 Teicoplanin from + 49 to + 60	44
#13	No	Ceftazidime from + 6 to + 9. Teicoplanin from + 7 to + 16. Ceftriaxone from + 9 to + 15. Piperacillin–Tazobactam from + 9 to + 25. Oxacillin from + 6 to + 23	20
#14	No	Piperacillin–Tazobactam from + 2 to + 9. Meropenem from + 9 to + 25. Linezolid from + 9 to + 13. Ceftazidime from + 57 to + 68. Ceftazidime from + 82 to + 91. Piperacillin–Tazobactam from + 91 to + 102. Daptomycin from + 91 to + 99	57
#15	No	Ceftazidime from + 2 to + 7. Levofloxacin from + 5 to + 7. Ceftriaxone from + 7 to + 9. Piperacillin–Tazobactam from + 9 to + 19. Teicoplanin from + 9 to + 19. Ceftazidime from + 30 to + 36	25
#16	Yes	Levofloxacin from − 9 to + 20. Ceftazidime from + 9 to + 20	30
#17	No	Piperacillin–Tazobactam from + 5 to + 6. Meropenem from + 6 to + 12. Ceftazidime from + 12 to + 16. Piperacillin–Tazobactam from + 16 to + 27. Teicoplanin from + 18 to + 36	32
#18	No	Ceftazidime from + 6 to + 15. Ceftazidime from + 36 to + 40. Teicoplanin from + 40 to + 43	18

Total days of antibiotics during hospitalization: Mean = 33.1 days; Median: 28 days (range 18–58 days). Total days of Piperacillin–Tazobactam during hospitalization: Mean = 10.6 days; Median: 8 days (range 0–33 days). Total days of Carbapenems during hospitalization: Mean = 5.2 days; Median: 0 days (range 0–25 days). *Non-SOS/VOD group*. Total days of antibiotics during hospitalization: Mean = 29.3 days; Median (range 12–57 days). Total days of Piperacillin–Tazobactam during hospitalization: Mean = 8.2 days; Median: 8.2 days (range 0–20 days). Total days of Carbapenems during hospitalization: Mean = 5.1 days; Median: 0 days (range 0–16 days). *Comparisons SOS/VOD vs non-SOS/VOD* (Mann–Whitney test). Total days of antibiotics during hospitalization: n.s. (P > 0.05). Total days of Piperacillin–Tazobactam during hospitalization: n.s. (P > 0.05). Total days of Carbapenems during hospitalization: n.s. (P > 0.05).

### GM profiling of SOS/VOD patients vs controls from before HSCT up to more than 2 months later

**Figure 1:**
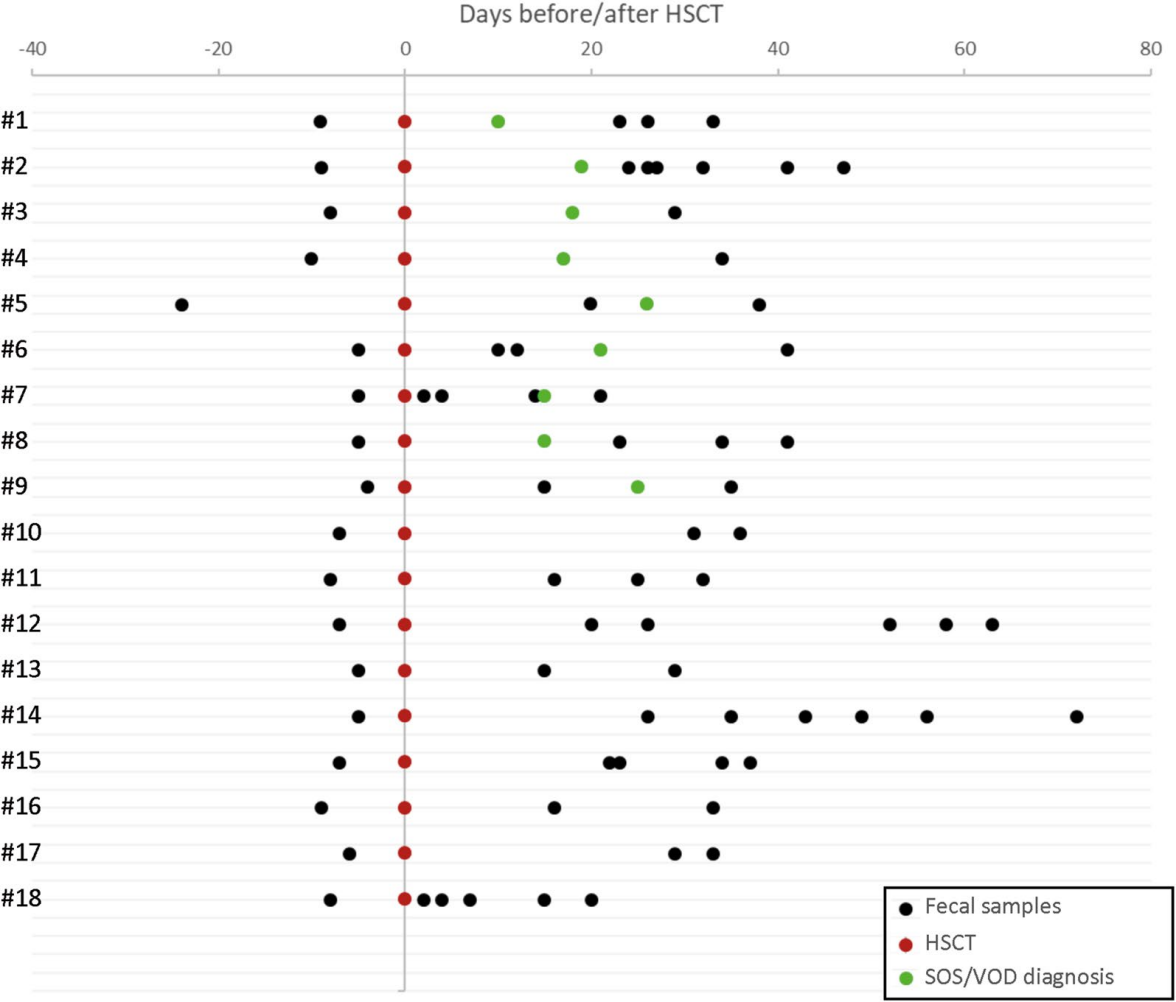
Schematic overview of the sampling time for each considered subject. HSCT (red dots), SOS/VOD diagnosis (green dots), and fecal sample collection (black dots) are plotted on timelines with distance from HSCT expressed in days.

A total of 74 fecal samples were available, collected before and after transplant, up to 72 days post-HSCT (Fig. 1). 16S rRNA gene sequencing of these samples yielded a total of 3,805,437 reads, ranging from 5246 to 233,603 per sample (mean 51,425, SD 39,961).

**Figure 2:**
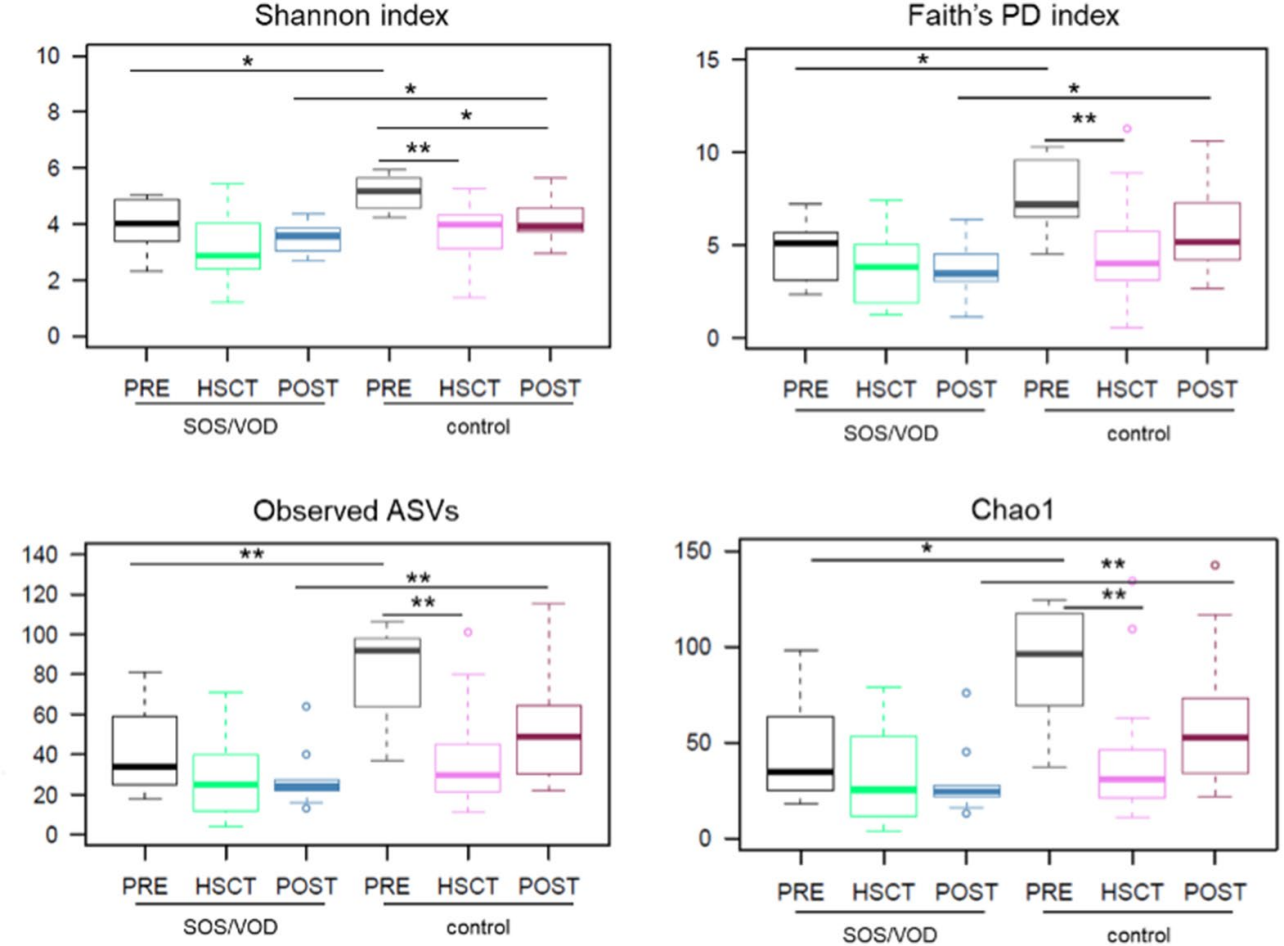
The gut microbiota of SOS/VOD patients before HSCT shows less diversity compared to control patients. Alpha diversity of fecal microbiota in samples taken before transplant (PRE), up to 30 days after transplant (HSCT) and more than 30 days after transplant (POST), calculated for SOS/VOD patients (on the left in each graph) and controls (on the right) using the following metrics: the Shannon index, the Faith’s PD index, the number of observed amplicon sequence variants (ASVs), and the Chao1 index. *P ≤ 0.05; **P ≤ 0.01; Wilcoxon test. See also Supplemental Figure s S1 and S2.

Pre-transplant samples (i.e., PRE samples) from patients who developed SOS/VOD showed significantly lower alpha diversity than controls, as measured by different metrics (i.e., the Shannon index, the number of observed ASVs, the Chao1 index, the Faith’s PD index, PE and AEDt index) (P ≤ 0.05, Wilcoxon test, false discovery rate (FDR) corrected). Consistent with the available literature^4,6^, during the first 30 days after HSCT, all subjects showed comparable low levels of GM diversity (HSCT samples). The diversity of post-transplant samples (POST samples) was again significantly lower in SOS/VOD-diagnosed patients than in controls (P ≤ 0.05), with the latter showing overall diversity levels comparable to those calculated for the PRE samples (Fig. 2 and Supplemental Figs. S1 and S2). A combined index of evenness and richness (inverse Simpson index) showed a similar trend, albeit in the absence of significance, with decreased average values in the PRE samples for patients who developed SOS/VOD compared to controls (Fig. 2 and Supplemental Fig. S1).

**Figure 3:**
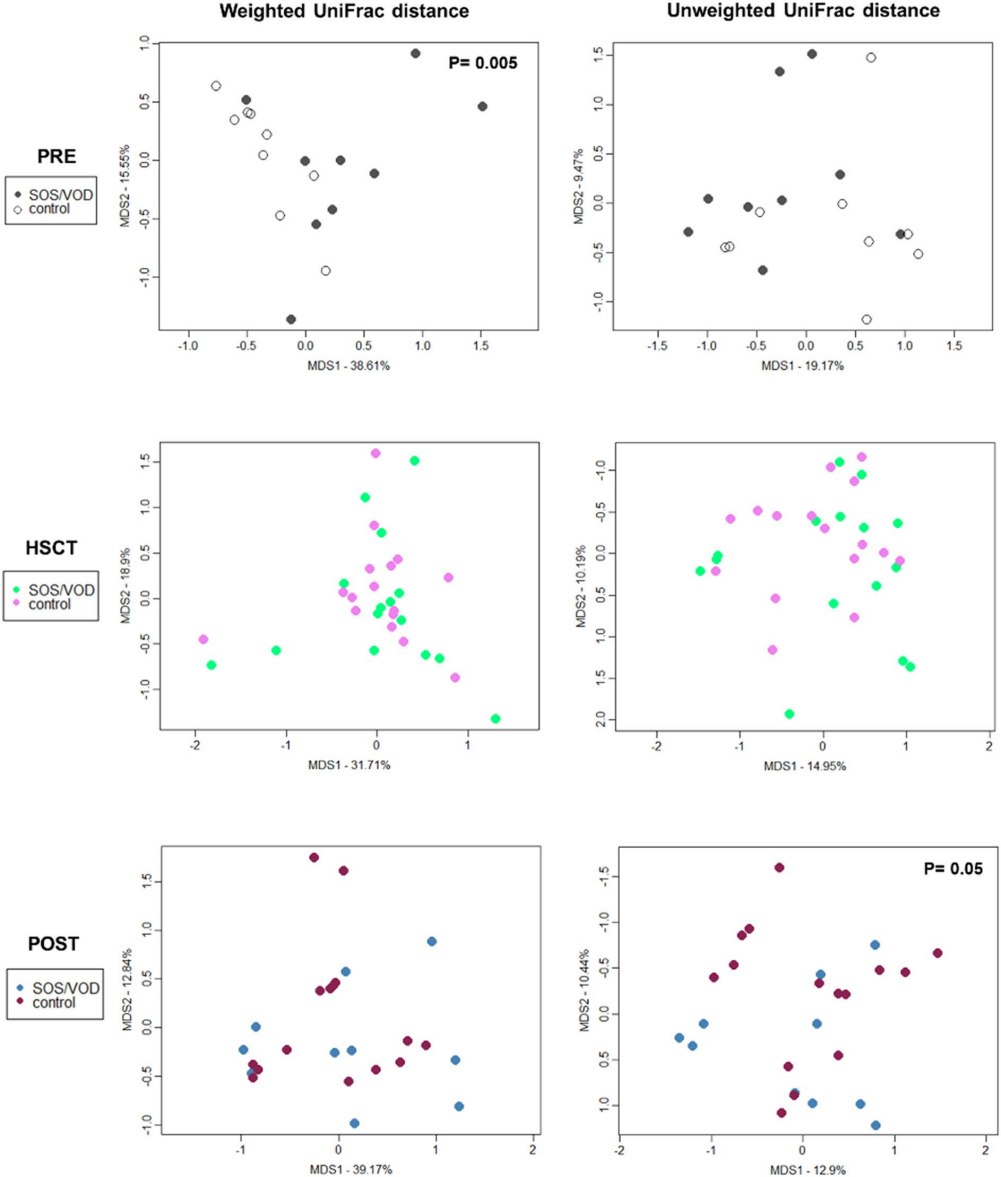
GM structure of SOS/VOD patients vs controls from before HSCT up to more than 2 months later. PCoA are based on weighted (left) and unweighted (right) UniFrac distances of microbiota profiles of samples collected (from top to bottom) before transplant (PRE), during the first 30 days after transplant (HSCT) and > 30 days after transplant (POST) from patients who subsequently developed SOS/VOD and controls (see the color legend on the left). First and second principal components (MDS1 and MDS2) are plotted for each analysis. Percentages of variance in the dataset accounted for by MDS1 and MDS2 are reported. Permutation tests with pseudo-F ratio (Adonis function in vegan package of R software) were carried on for each analysis to test the significance of separation between patients and controls; P values ≤ 0.05 are reported (top left in each plot).

Weighted and unweighted UniFrac distances between GM profiles were then computed and used to construct PCoA plots for each of the three defined time intervals, i.e., PRE, HSCT and POST. As shown in Fig. 3, PRE samples from SOS/VOD patients and controls significantly separated in the weighted UniFrac-based PCoA (P = 0.005, permutation test with pseudo-F ratio), confirming that early compositional differences might characterize the GM of pediatric patients who subsequently develop SOS/VOD. An additional analysis with PRE samples revealed no difference in the GM structure in relation to the administration of antibiotic prophylaxis (Supplemental Fig. S3).

Similarly to what is described above for alpha diversity, no significant difference was found within the first 30 days after HSCT, with the samples from SOS/VOD patients and controls overlapping in the PCoA space (Fig. 3). On the other hand, in the POST, i.e., beyond 30 days from HSCT, the two groups were again significantly separated in the PCoA using the unweighted UniFrac metric (P = 0.05), likely suggesting the persistence of some structural changes in low-abundance taxa.

**Figure 4:**
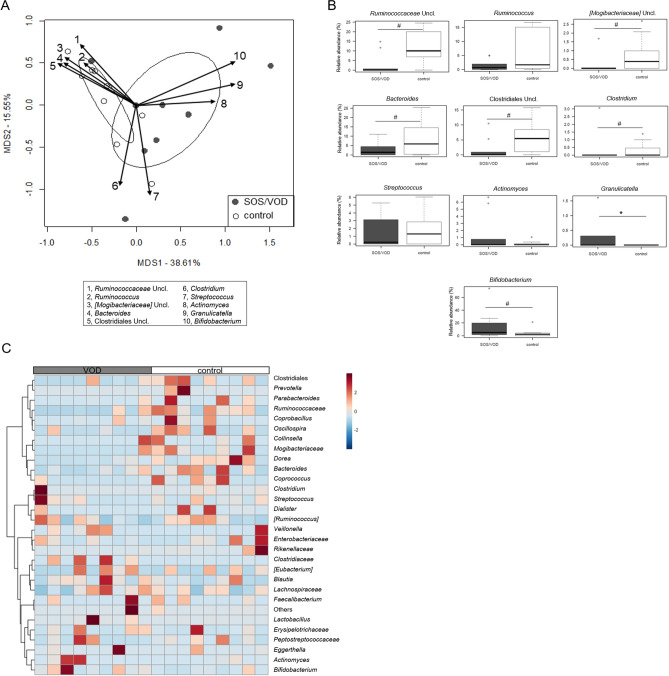
The gut microbiota of SOS/VOD patients before HSCT shows a different compositional structure compared to control patients. (**A**) PCoA based on weighted UniFrac distances between microbiota profiles of samples collected before transplant from patients who subsequently developed SOS/VOD (dark grey circles) and controls (empty white circles). First and second principal components (MDS1 and MDS2) are plotted, accounting for 38.61% and 15.55% of variance in the dataset, respectively. The separation between the two groups of samples (Adonis P = 0.008) is highlighted by plotting the SEM-based ellipse around the centroid. The biplot of the average bacterial coordinates weighted by the corresponding relative abundance per sample was superimposed on the PCoA plot for the 10 genera significantly contributing to the ordination space, as calculated using the function envfit in the vegan package of R (black arrows). The number associated with each arrow identifies the bacterial group contributing to the ordination space; the legend is shown at the bottom. (**B**) Boxplots showing the relative abundance distribution of taxa identified with envfit function in SOS/VOD and control groups. *P ≤ 0.05; ^#^0.05 < P ≤ 0.1; Wilcoxon test. *Uncl. *unclassified. (**C**) Heatmap showing Ward-linkage clustering based on the Pearson’s correlation coefficients of the relative abundance of genus-level taxa from SOS/VOD patients (see the grey bar at the top) and controls (white bar) before HSCT. Only taxa with relative abundance > 0.1% in at least 1 sample are shown. Unclassified genera are reported at higher taxonomic level. See also Fig. 5 and Supplemental Figure S4.

In order to identify discriminating taxa between patients who did or did not develop SOS/VOD, we plotted the bacterial genera contributing most to the ordination space, using the function envfit in the vegan package of R (P ≤ 0.05). It was therefore possible to ascertain that the PRE samples from control subjects were characterized by the *Bacteroides* genus and unclassified bacteria belonging to the Clostridiales, *Ruminococcaceae* and [*Mogibacteriaceae*] groups (Fig. 4A). The comparison of the relative abundances of these taxa in the two subject groups allowed to confirm the observed trends (P ≤ 0.1, Wilcoxon test) (Fig. 4B). Furthermore, the trend of *Bacteroides* was confirmed by qPCR (Supplemental Fig. S4).

In contrast, no clearly defined groups of bacteria appeared to characterize patients who subsequently developed SOS/VOD, whose samples were actually more dispersed in the PCoA plot, indicating greater inter-sample variability. However, it is worth noting that *Granulicatella* (P = 0.03) and *Bifidobacterium* (P = 0.1) were significantly more represented or tended to be, in the PRE samples belonging to SOS/VOD patients (Fig. 4). This was likely related to the presence in this group of two patients aged less than 2 years, in line with the available literature on the infant-type GM^32,33^*.* Furthermore, some patients shared relatively high proportions of *Actinomyces*, as well as *Erysipelotrichaceae* and *Peptostreptococcaceae* genera (Fig. 4C), which are commonly overrepresented in inflammation-related disorders^34,35^. A subgroup analysis by removing the samples corresponding to the infants from both SOS/VOD patients and controls, confirmed the main findings of this study (Supplemental Fig. S5), thus suggesting that previously identified microbiota signatures may be age independent.

**Figure 5:**
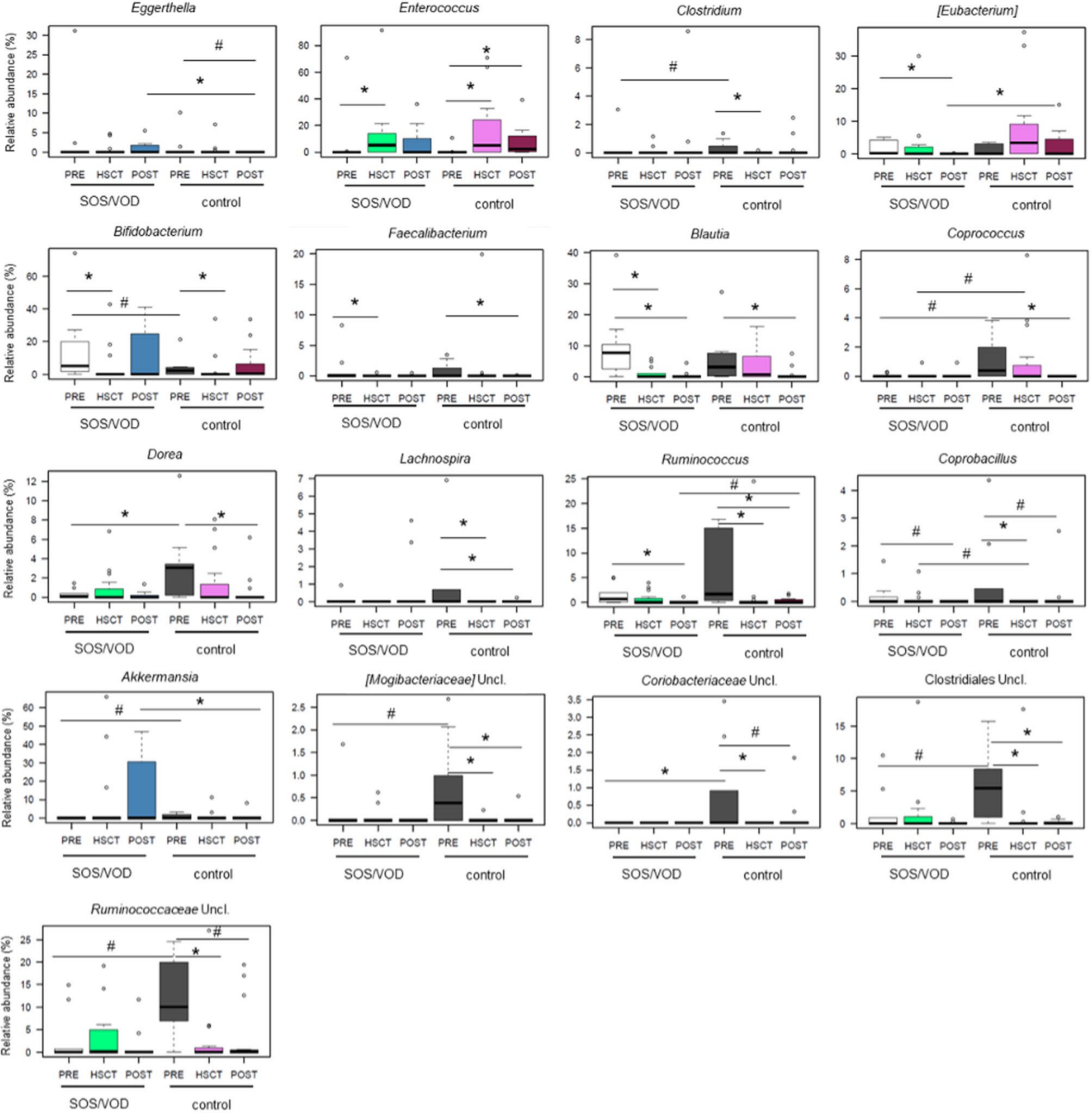
Gut microbiota compositional changes in SOS/VOD patients vs controls from before HSCT up to more than 2 months later. Boxplots showing the relative abundance distribution of genera differentially represented between and within groups. Dynamics of each genus were reconstructed from before transplant (PRE), during the first 30 days after transplant (HSCT) up to > 30 days after transplant (POST), for patients who subsequently developed SOS/VOD (on the left in each graph) and controls (on the right). *P ≤ 0.05; ^#^0.05 < P ≤ 0.1; Wilcoxon test. *Uncl*. unclassified.

Finally, we evaluated the GM compositional changes in the whole cohort, i.e., within and between groups over time (Fig. 5). Notably, we found that the PRE samples from SOS/VOD patients were also characterized by depletion in other typically health-associated taxa, such as *Lachnospiraceae* genera (i.e., *Coprococcus* and *Dorea*) and *Akkermansia*, as well as in unclassified members of *Coriobacteriaceae* (P ≤ 0.1). Furthermore, the opportunistic pathogen *Eggerthella* was overrepresented in the POST samples of the SOS/VOD patients compared to the controls, while the opposite was observed for [*Eubacterium*] (P ≤ 0.05). Although the decrease over time in beneficial taxa, such as *Blautia*, *Faecalibacterium* and *Ruminococcus*, was shared by both groups of subjects, SOS/VOD patients showed generally lower proportions or faster decreases (e.g. already in the HSCT samples for *Blautia* and *Faecalibacterium*) (P ≤ 0.1). The data on the differential trends of members of the *Lachnospiraceae* and *Ruminococcaceae* families were confirmed by qPCR (Supplemental Fig. S4).

## Discussion

### Potential protective GM signature against SOS/VOD

This pilot study reports for the first time a possible association between GM and the onset of SOS/VOD. Well aware of the impossibility of postulating any causal relationship, here we advance the hypothesis that a rich and diverse GM before HSCT could be associated with a decreased probability of developing SOS/VOD. This observation is consistent with the recent scenario in which a high level of GM diversity, a cornerstone of a so-called “healthy” GM, has been associated with decreased mortality after HSCT^4^. Greater alpha diversity is linked as well to a reduced likelihood of developing another post-HSCT complication, aGvHD in pediatric patients^6^. The protective signature identified, rich in *Bacteroides*, *Lachnospiraceae* genera and unclassified members of Clostridiales and *Ruminococcaceae,* also shares some similarity with the protective configuration observed in studies regarding aGvHD. In particular, other authors have previously shown that higher relative abundances of *Lachnospiraceae* and *Ruminococcus* were associated with better clinical outcomes, mainly reduced aGvHD incidence and severity^36,37^. As briefly discussed above, the presence of *Bifidobacterium* in two patients who went on developing SOS/VOD could be partially explained by their age, these being children under the age of two and breastfed^38^. Similarly, *Granulicatella*, which was more represented in SOS/VOD patients, has been identified as a signature of the infant gut microbiota, probably due to its involvement in lactate metabolism^33^. However, some species have been recognized as possible agents of bloodstream and endovascular infections, especially when predisposing conditions such as mucositis and neutropenia are present^39,40^, thus potentially representing a “red flag” for clinicians. The protective effect exerted by the peculiar pre-HSCT GM composition in both SOS/VOD and aGvHD could be related to the common endothelial damage that characterizes both diseases. As for the other complications, the mechanistic explanation for this effect remains poorly understood. Based on previous preclinical studies^9,10^, we hypothesize that an altered intestinal ecosystem, depleted of health-associated taxa and with low production of SCFAs, could lead to greater translocation of microbial molecules through the damaged intestinal mucosa. Consistent with this, the proportions of the most common SCFA producers (from *Lachnospiraceae* and *Ruminococcaceae* families) remained overall lower in SOS/VOD patients than controls, even over time. Translocated bacterial products, and in particular LPS, could reach the liver sinusoid through the portal vein and participate in endothelial damage^9,10^. It is also worth mentioning that *Bacteroides*, found to be enriched in the control group, is one of the major producers of propionate, which has been shown to induce vasodilatation ex vivo and in animal models^14^, and appears to lack the potential to produce trimethylamine^41^, precursor of TMAO, which is well known to be involved in vasculo-occlusive events^12^. However, since metabolic profiles were not evaluated in this study, future investigations will be mandatory to test our hypotheses and clarify which functional players are actually involved.

### Analysis of confounding variables

This study has some limitations, mainly due to its retrospective design. The number of patients is relatively small because of the low incidence of this complication. The GM sequences obtained in this study were combined with sequences from previous studies, but all samples were collected by the authors, processed in the same laboratory and thus subjected to the same wet and in silico analysis steps. Moreover, the timing of fecal sample collection, which depends on patients’ bowel movements, that in children undergoing HSCT are particularly irregular, is not fully matched between the two groups. To address this possible confounding variable, we performed a diversity analysis using only one sample per subject in each time window and the results were very similar to those found considering all samples, i.e., the patients who developed SOS/VOD had a less diverse GM in the pre and post-HSCT samples compared to the control patients (Supplemental Fig. S2). As for the relative imbalance in the administration of antibiotics between the two study groups, no difference was found in the total antibiotic exposure and in the administration of specific antimicrobial molecules (Table 2). Moreover, a subgroup analysis to better address the imbalance of levofloxacin prophylaxis between the two groups, revealed no separation in the PRE GM samples between patients who did or did not receive levofloxacin prophylaxis (Supplemental Fig. S3).

In conclusion, our pilot study’s findings stress the relevance of having a GM characterized by high diversity and richness of beneficial microorganisms in the pre-transplant period, as it could be associated with a reduced occurrence of SOS/VOD, as well as other HSCT-related complications and overall survival. The present results should prompt additional studies to better investigate the possible interplay between GM dysbiosis, endothelial cell injury and alloreactivity occurring in the pathogenesis of SOS/VOD, also using other techniques, including metabolomics. Studies involving larger cohorts of patients, possibly of various age groups to account for age-related specificities of the GM, are warranted to explore whether GM structure and functionality could serve as a predictive biomarker for clinicians to assess the risk, onset and progression of SOS/VOD. Such studies should include, as far as possible, a finer sampling even in the post-HSCT period, to define healthy/unhealthy trajectories of the gut microbiota across HSCT. This study also corroborates to ensure scientific bases for future GM-based interventions, such as fecal microbiota transplantation, to assess whether GM manipulation may impact on the incidence of HSCT complications.

## Supplementary Information

Supplementary Information.

## Data Availability

Sequencing reads from samples of patients 5, 8, 9 and 16 are available at http://www.ebi.ac.uk/ena/data/view/PRJEB23820 (corresponding to subjects B2, B1, B3 and B5, respectively; Biagi et al.^6^). Sequencing reads from samples of patients 1, 2, 11–15 are available at https://www.ncbi.nlm.nih.gov/bioproject/PRJNA592853 (corresponding to patients BP9, BE4, BP10, BE3, BP4, BE6 and BP6; D’Amico et al.^18^). Sequencing reads from newly sequenced samples (from patients 3, 4, 6, 7, 10, 17 and 18) are available at https://www.mg-rast.org/linkin.cgi?project=mgp97083, labelled as patients BE19, BP11, BE14, BE15, BE13, BE12 and BE16, respectively.
