# Supplementary material for: Early modifications of the gut microbiome in children with hepatic sinusoidal obstruction syndrome after hematopoietic stem cell transplantation
Source: Sci Rep. 2021 Jul 12;11:14307. doi: 10.1038/s41598-021-93571-4 (PMC8275574; doi:10.1038/s41598-021-93571-4)
Supplement: Supplementary file 1 — Supplementary Information. [file 41598_2021_93571_MOESM1_ESM.pdf]

Early modifications of the gut microbiome in children with hepatic sinusoidal obstruction syndrome after hematopoietic stem cell transplantation

Riccardo Masetti[1,3], Elena Biagi[2], Daniele Zama[1], Edoardo Muratore[1], Federica D'Amico[2,3], Davide Leardini[1*], Silvia Turroni[2], Arcangelo Prete[1], Patrizia Brigidi[3], Andrea Pession[1,3]

[1] Pediatric Oncology and Hematology "Lalla Seràgnoli", Pediatric Unit, IRCCS Azienda Ospedaliero-Universitaria di Bologna, 40138 Bologna, Italy;

[2] Unit of Microbiome Science and Biotechnology, Department of Pharmacy and Biotechnology, University of Bologna, 40126 Bologna, Italy;

[3] Department of Medical and Surgical Sciences (DIMEC), University of Bologna, 40138 Bologna, Italy;

[*] Correspondence: davide.leardini3@studio.unibo.it, Tel.: +39-051-214-4665, Pediatric Oncology and Hematology "Lalla Seràgnoli", Pediatric Unit, IRCCS Azienda Ospedaliero-Universitaria di Bologna, 40138 Bologna, Italy.

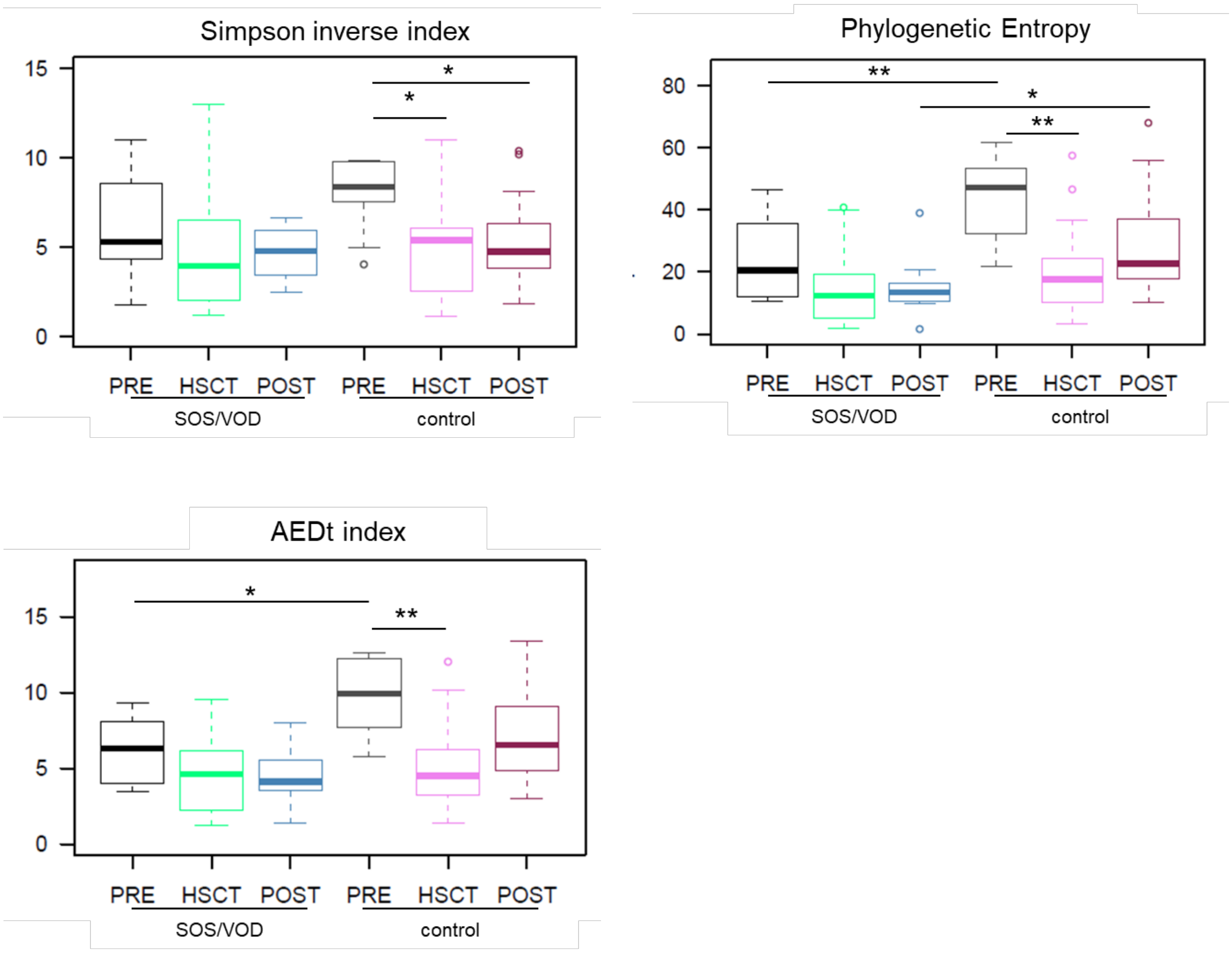

Supplemental Figure S1. Alpha diversity of the gut microbiota of SOS/VOD patients compared to control patients. Alpha diversity in samples taken before transplant (PRE), up to 30 days after transplant (HSCT) and more than 30 days after transplant (POST), calculated for SOS/VOD patients (on the left in each graph) and controls (on the right) using the following metrics: the inverse Simpson index, phylogenetic entropy (PE) and the abundance weighted evolutionary distinctiveness (AEDt) index. *, $P \leq 0.05$; **, $P \leq 0.01$; Wilcoxon test. See also Figure 2.

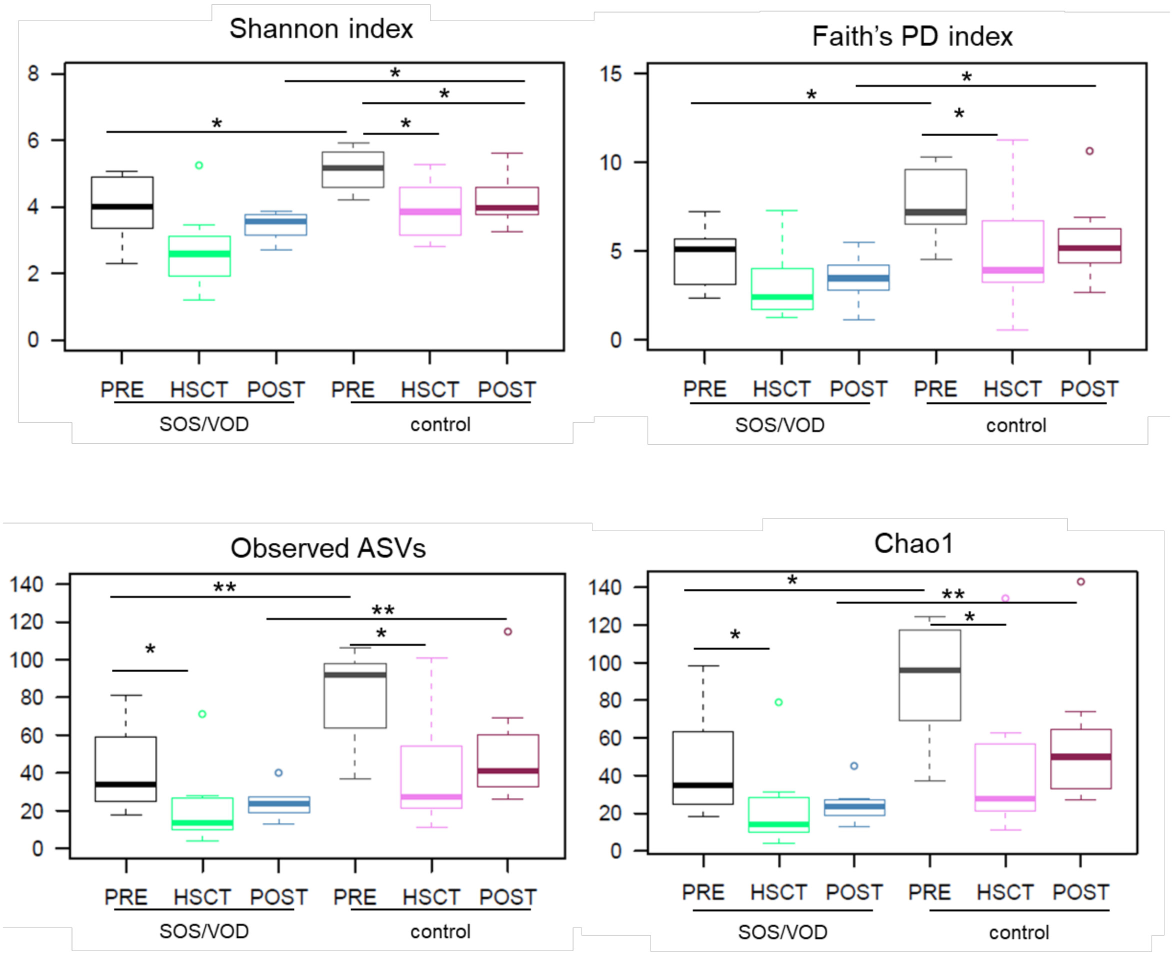

Supplemental Figure S2. Analysis of alpha diversity using one sample for each time window. Alpha diversity of fecal microbiota in samples taken before transplant (PRE), up to 30 days after transplant (HSCT) and more than 30 days after transplant (POST), calculated for SOS/VOD patients (on the left in each graph) and controls (on the right) using the following metrics: the Shannon index, the Faith's PD index, the number of observed amplicon sequence variants (ASVs), and the Chao1 index. *, P ≤ 0.05; **, P ≤ 0.01; Wilcoxon test. See also Figure 2.

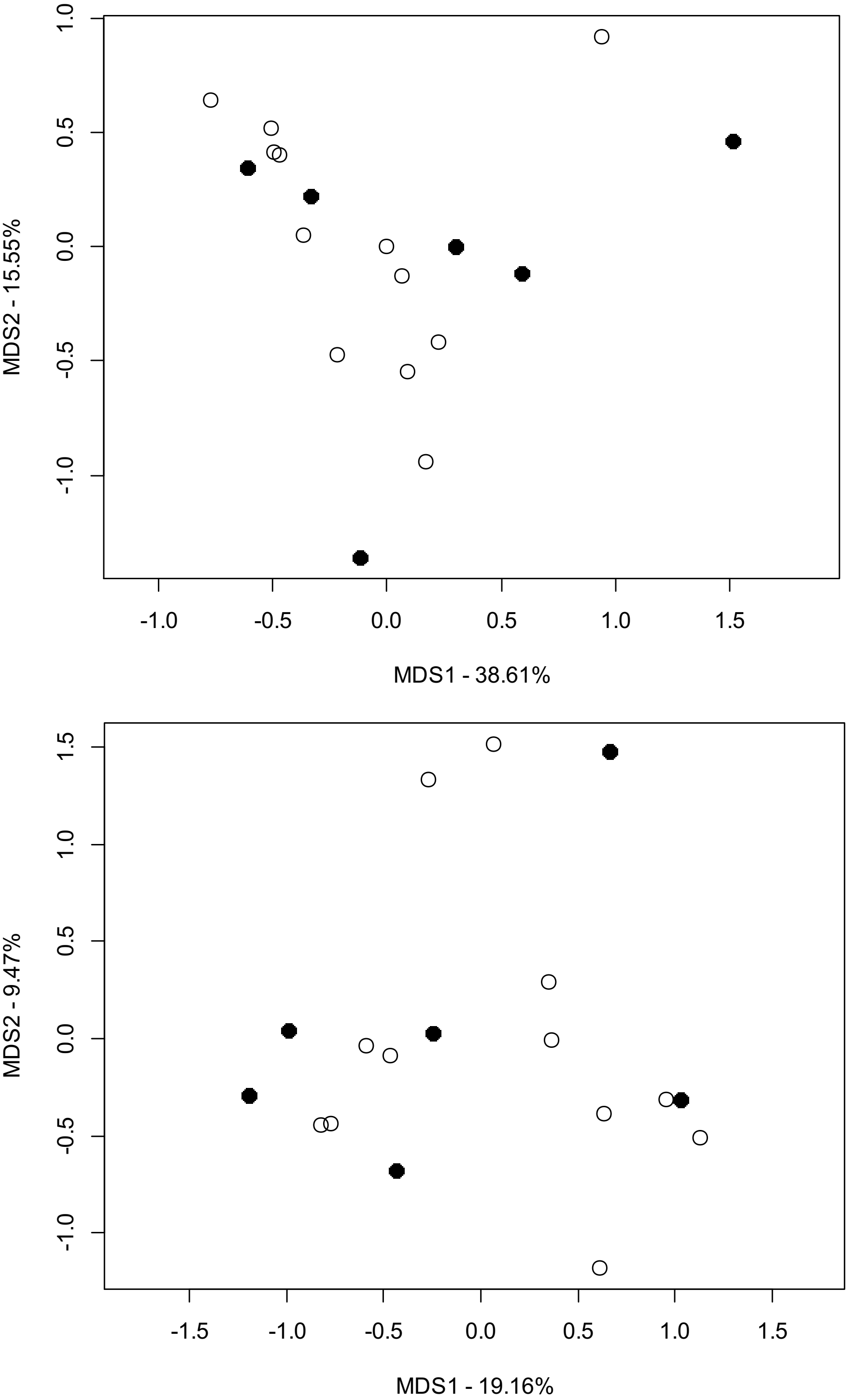

Supplemental Figure S3. Analysis of levofloxacin prophylaxis contribution. PCoA plots based on weighted (top) and unweighted (bottom) UniFrac distances of microbiota profiles of samples collected before transplant (PRE) from patients who received (black circles) and did not receive (empty white circles) levofloxacin prophylaxis prior to HSCT. First and second principal components (MDS1 and MDS2) are plotted for each analysis. Percentages of variance in the dataset accounted for by MDS1 and MDS2 are reported. Permutation tests with pseudo-F ratio (Adonis function in vegan package of R software) were carried on for each analysis to test the significance of the separation between patients and control samples; P values > 0.05 were obtained for both distance metrics (P = 0.46 and P= 0.63, for weighted and unweighted UniFrac distances, respectively).

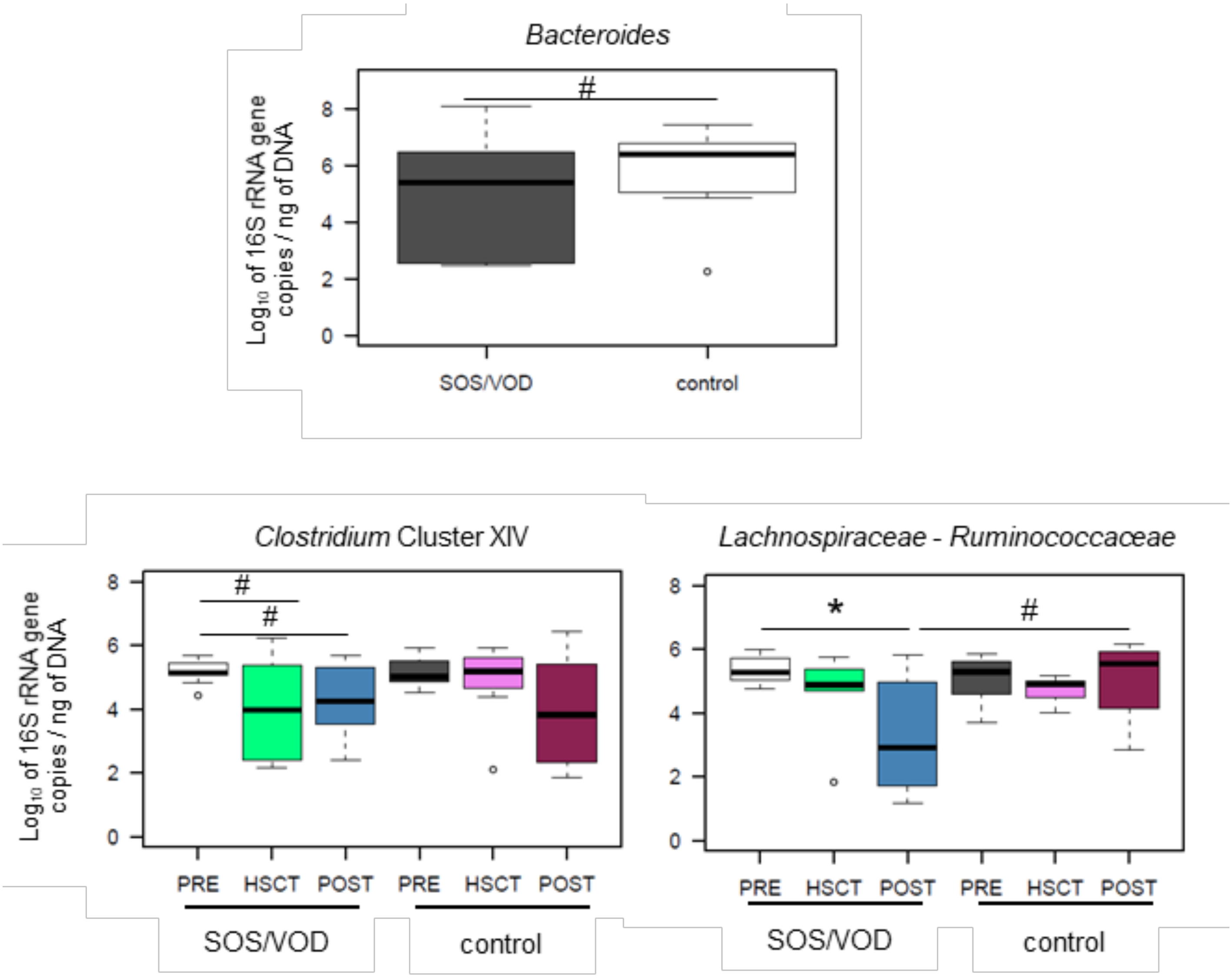

Supplemental Figure S4. The reduction in the proportions of typically health-associated taxa in SOS/VOD patients was confirmed by qPCR. Boxplots showing the distribution of log10 16S rRNA gene copies per ng of DNA of *Bacteroides* (top) and *Lachnospiraceae* and *Ruminococcaceae* genera (bottom) in SOS/VOD patients (on the left in each graph) and controls (on the right). For *Bacteroides*, only the comparison before transplant is shown. For *Lachnospiraceae* and *Ruminococcaceae* genera, two primer sets, targeting *Clostridium* cluster XIVab and *Lachnospiraceae-Ruminococcaceae,* were used (see Methods). Samples were taken before transplant (PRE), up to 30 days after transplant (HSCT) and more than 30 days after transplant (POST). *, P ≤ 0.05; #, 0.05 < P ≤ 0.1; Wilcoxon test.

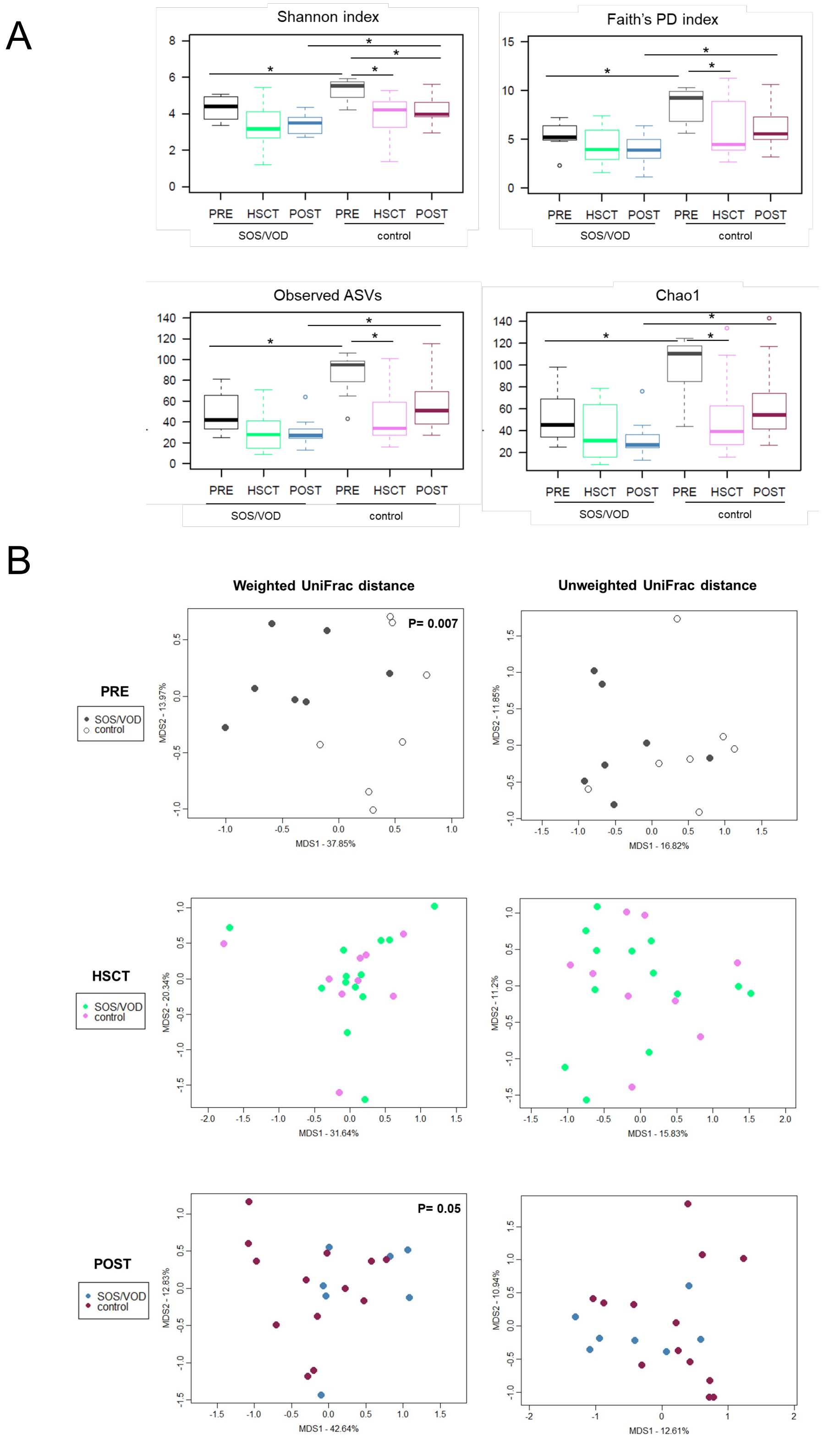

Supplemental Figure S5. Subgroup analysis of gut microbiota diversity of SOS/VOD patients compared to controls excluding infants. A, Alpha diversity in samples taken before transplant (PRE), up to 30 days after transplant (HSCT) and more than 30 days after transplant (POST), calculated for SOS/VOD patients (on the left in each graph) and controls (on the right) using the following metrics: the Shannon index, the Faith's PD index, the number of observed amplicon sequence variants (ASVs), and the Chao1 index. *, P ≤ 0.05, Wilcoxon test. B, PCoA plots based on weighted (left) and unweighted (right) UniFrac distances of microbiota profiles of PRE, HSCT and POST samples (from top to bottom) from patients who subsequently developed SOS/VOD and controls (see the color legend on the left). Permutation tests with pseudo-F ratio (Adonis function in vegan package of R software) were carried on for each analysis to test the significance of separation between patients and controls; P values ≤ 0.05 are reported (top left in each plot). Two infant patients were excluded from each group.

Confounding variables analysis. The distribution of possible confounding variables (Graft versus Host Disease, GvHD; Blood Stream Infection, BSI; levofloxacin administration, LVX; Enteral or Parenteral nutrition during the first weeks after HSCT) among the enrolled patients was explored. Fisher exact test was used on contingency tables (**Supplemental Tables S1-S4,** values indicate the number of patients for each variables combination). None of the four possible confounding variables examined was significantly associated with the SOS/VOD occurrence (Fisher test, $P > 0.05$). Levofloxacin prophylaxis as a possible confounding variable impacting on the pre-transplant fecal microbiome composition was also specifically explored in Supplemental Figure S3.

Supplemental Table S1

	GvHD	No GvHD
SOS/VOD	3	6
No SOS/VOD	5	4

Supplemental Table S2

	BSI	No BSI
SOS/VOD	1	8
No SOS/VOD	4	5

Supplemental Table S3

	LVX	No LVX
SOS/VOD	4	5
No SOS/VOD	1	8

Supplemental Table S4

	Enteral	Parenteral
SOS/VOD	4	5
No SOS/VOD	5	4
